# Incidence of histoplasmosis in patients receiving TNF-alpha inhibitors: A systematic review and meta-analysis

**DOI:** 10.1097/MD.0000000000036450

**Published:** 2023-12-08

**Authors:** Murillo M. Cipolat, Débora R.R. Rodrigues, Claiton V. Brenol, Alessandro C. Pasqualotto, Diego R. Falci

**Affiliations:** a Graduate Program in Medical Sciences (PPGCM), Universidade Federal do Rio Grande do Sul, Porto Alegre, Brazil; b Santa Casa de Misericórdia de Porto Alegre, Porto Alegre, Brazil; c Universidade Federal de Ciências da Saúde de Porto Alegre, Porto Alegre, Brazil; d Pontifícia Universidade Católica do Rio Grande do Sul, Porto Alegre, Brazil; e Hospital de Clinicas de Porto Alegre, Porto Alegre, Brazil.

**Keywords:** Anti-TNF-α, Histoplasmosis, Inflammatory Bowel Disease, Rheumatoid Arthritis, TNF-ɑ inhibitors

## Abstract

**Background:**

Immunobiological drugs such as TNF-α inhibitors are valuable in rescue therapy for autoimmune diseases such as rheumatoid arthritis and inflammatory bowel disease (IBD), but they increase the risk of infectious complications. Histoplasmosis is a significant concern in patients living in endemic regions, however, few studies have assessed the incidence of *Histoplasma* infection during therapy, and classic estimates may underestimate the risk. This study aimed to produce an updated risk estimate of histoplasmosis in patients on TNF-α blocking therapy.

**Methods:**

This is a systematic review and meta-analysis of studies that contain parameters for calculating the risk of histoplasmosis in people who use TNF-α inhibitors, to produce a risk estimate.

**Results:**

We identified 11 studies with the necessary parameters for inclusion in the meta-analysis, most of which were from North America. The incidence rate of histoplasmosis found was 33.52 cases per 100,000 patients treated with TNF-ɑ inhibitors (95% CI 12.28–91.46). Considering only studies evaluating monoclonal antibodies, the calculated incidence was 54.88/100,000 patients treated (95%CI 23.45–128.34). In subgroup analysis, the incidence was much higher in patients with IBD compared to rheumatic diseases. There was significant heterogeneity among the studies.

**Conclusion:**

The risk of histoplasmosis during TNF-α inhibitory therapy may be considerably higher than that found in classical estimates, especially in patients with IBD. There is a lack of studies evaluating histoplasmosis in large endemic areas, such as Central and South America.

## 1. Introduction

Endemic fungal diseases represent a heterogeneous group of neglected infections that are generally characterized by limited diagnostic and therapeutic options, especially in developing countries. In this category, histoplasmosis is considered endemic in the United States (US) Midwest and many regions of Central and South America, as well as in Asia and Africa.^[1–3]^ Histoplasmosis is the disease caused by the dimorphic fungus *Histoplasma capsulatum*, which can range from an asymptomatic infection to a systemic disease that, in the disseminated form, has an important possibility of progressing to death. The disease is regarded as one of the most common endemic mycoses in HIV-infected patients in those areas, causing high morbidity and mortality.^[4]^ Ninety-five percent (95%) of patients with acquired immunodeficiency syndrome (AIDS) and *H capsulatum* infection develop disseminated disease, with 90% having CD4 counts below 200 cells/mm^3^ at the time of diagnosis.^[5]^ In the US, state-specific annual incidence rates ranged from 0 to 4.3 cases/100,000 population, with the highest rates found in cities in endemic areas, including the Ohio and Mississippi River valleys.^[6]^

The treatment of inflammatory autoimmune diseases, such as rheumatoid arthritis (RA), inflammatory bowel disease (IBD), psoriasis, psoriatic arthritis, and ankylosing spondylitis, has made substantial progress with the development of immunobiological therapies, especially in cases refractory to treatment with traditional medications. Among the first drugs of this group to be incorporated into clinical practice are the tumor necrosis factor-alpha inhibitors (TNF-ɑ inhibitors, also called Anti-TNF-ɑ), approved by the Food and Drug Administration (FDA) in the 1990s.^[7–11]^ However, even though they are considered fundamental in rescue therapy for refractory diseases, they have great potential to block the immune response necessary against infections.^[12–15]^ Thus, patients using these drugs are at an increased risk of opportunistic infections, notably tuberculosis.^[16]^

Among fungal infections, histoplasmosis is considered the most frequent opportunistic infection in patients using TNF-α inhibitors in endemic areas.^[17]^ The widespread use of these therapies in the US has resulted in a significant increase in hospitalizations for this disease in histoplasmosis endemic regions.^[18]^ Like AIDS patients, mortality can be high, which can be a complication of delayed diagnosis.^[17]^ In addition, the frequency of histoplasmosis may vary depending on the different TNF-α inhibitors, concomitant use of other immunosuppressive agents (e.g., corticosteroids), and the type of autoimmune diseases.^[19]^

Despite the large number of patients using TNF-α inhibitors in clinical practice, there is still no systematic review of the medical literature regarding the incidence of histoplasmosis in this population. Therefore, in this study, we performed a systematic review of data available over the last 25 years to clarify the risk of histoplasmosis as a complication of treatment with TNF-α inhibitors for autoimmune diseases.

## 2. Methods

### 2.1. Data search

Eligible studies were identified through search of Pubmed MEDLINE, Embase and Cochrane Library databases, by 2 researchers independently, from insertion of the following combination of text-words: (Tumor Necrosis Factor Inhibitors[mh] OR TNF Inhibitor* OR TNF-alpha inhibitor* OR Anti-TNF*[tw]) AND (Histoplasmosis[mh]), (Rheumatoid arthritis[mh] OR inflammatory bowel disease[mh]) AND (Histoplasmosis[mh]), (Rheumatoid arthritis[mh] OR inflammatory bowel disease[mh]) AND (mycosis[mh] OR fungal infections[mh]), (Rheumatoid arthritis[mh] OR inflammatory bowel disease[mh]) AND (invasive fungal infection[mh]), (Tumor Necrosis Factor Inhibitors OR Anti-TNF) AND (mycosis[mh] OR fungal infections[mh]), and (Immunosuppressive Drugs OR Immunosuppressants[mh]) AND (Histoplasmosis[mh]). Articles published from 1998 (the year of FDA approval of the first TNF-α inhibitor-type drug) to February 2023 were selected. The references of the selected articles were also reviewed. Although multiple conditions can be treated with TNF-α inhibitory drugs, RA and IBD have been highlighted because most large population studies address these 2 pathologies.

### 2.2. Study selection

Studies involving human subjects published during the specified period were considered eligible for inclusion. Articles that contained case series of patients diagnosed with histoplasmosis during therapy with TNF-α inhibitor agents or reports that reviewed the infectious complications of the biological therapies under analysis, including product safety reports, were selected for complete text analysis. The selection sought articles that allow a calculation involving a population as the denominator—a group of patients being treated with TNF-α inhibitors—and a numerator—patients from this population diagnosed with histoplasmosis during the treatment. All studies containing the specified data were included in the final analysis of results. Publications dealing with single case reports and studies on non-human histoplasmosis models were not included in the review.

### 2.3. Outcome of interest

**Figure 1.**
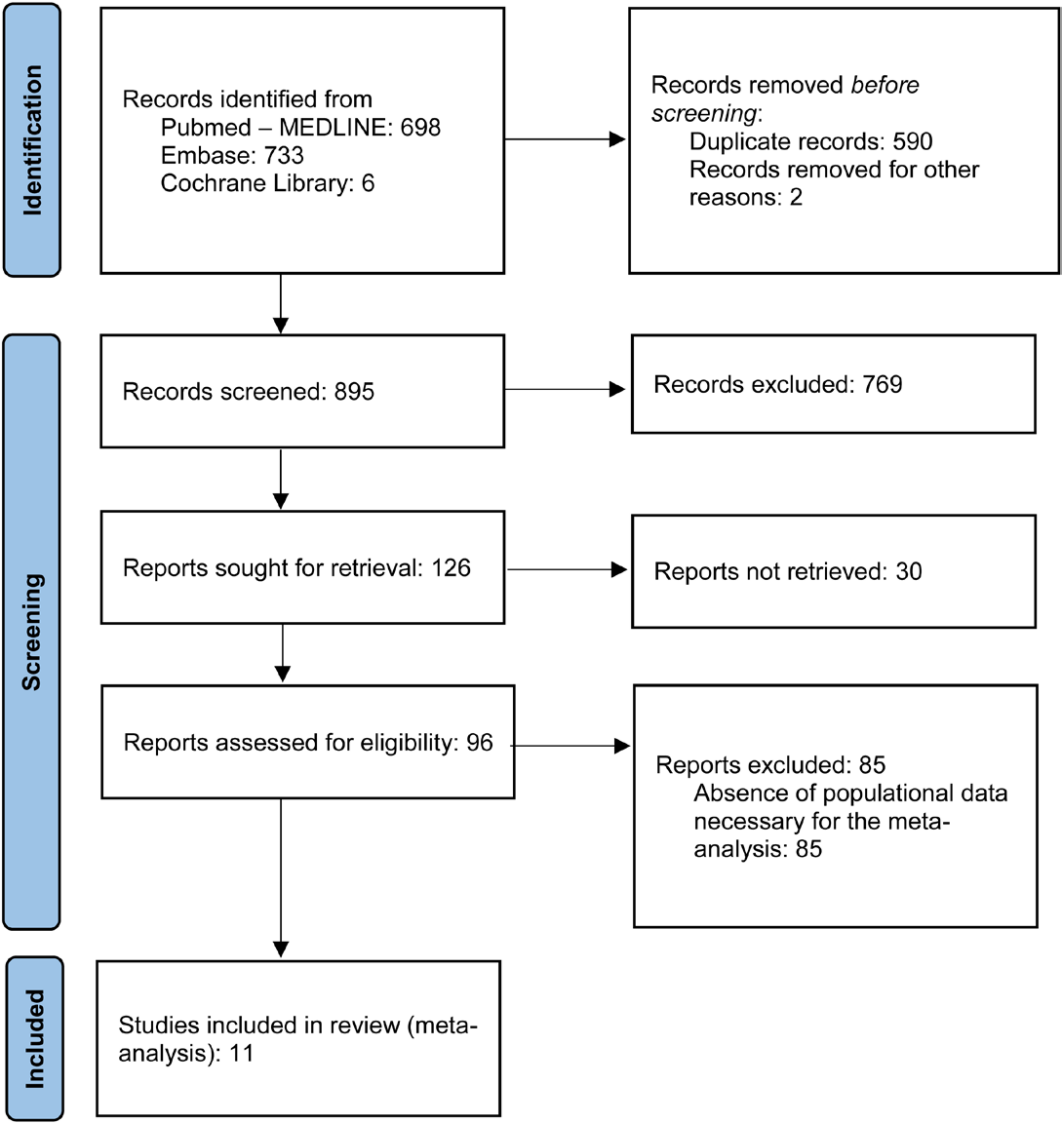
Flow diagram for the systematic review of databases and records on the incidence of histoplasmosis in patients receiving TNF-alpha inhibitors.

This systematic review aims to bring together studies in which the incidence rate of histoplasmosis as an opportunistic fungal disease occurring in a population undergoing a specific treatment, TNF-α inhibitors as a medication for autoimmune diseases, can be obtained. While some of the selected studies (such as the large epidemiological studies) already presented this data, in others we calculated the incidence rate based on 2 parameters: the total number of people using TNF-α inhibitors evaluated in each study and the number of these patients who were diagnosed with histoplasmosis during follow-up. Finally, a meta-analysis was performed by aggregating the data provided by the studies that met the inclusion criteria to calculate the incidence rate of histoplasmosis in the study population. A flow diagram illustrating the search strategy used is available in Figure 1.

### 2.4. Statistical analysis and ethical aspects

The software used for the quantitative analysis was the R-Project *version 4.2.0*, meta package (*version: 5.2-0 command: metaprop*). Considering the 95% confidence intervals (95% CI) of the incidence rate of each study, the heterogeneity of the articles included in the meta-analysis was evaluated by the Cochran Q test, and the inconsistency test (i²) was calculated. Due to the variation among the populations studied, the summarizing measures (incidence) were calculated using the random effects model. Subgroup analysis was performed according to each disease group (rheumatic diseases, IBD) when this information was specified. In addition, a sensitivity analysis was performed, removing studies that used only etanercept (two articles). The significance level was set at *P* value of < .05. The project was approved by the Ethics Committees of the institutions in which the authors of this study participate.

## 3. Results

### 3.1. Literature search

Using the search strategy, after eliminating duplicate records and combining the collections (MEDLINE, Embase, and Cochrane Library), 895 results were obtained. After analyzing the abstracts, 96 studies were selected for the full-text analysis. After this step, 11 articles were selected because they contained the necessary information for analysis of the outcome of interest.^[20–30]^ The selected studies and their main characteristics are listed in Table 1.

**Table 1:** Studies included in the systematic review and meta-analysis.

Study publication title	Year of publication	Study design	Main findings
Wallis RS et al Reactivation of latent granulomatous infections by infliximab^[20]^	2005	Review on Granulomatous Infections in patients on Infliximab, in which the authors compared the number of cases of granulomatous infections in US patients treated with infliximab or etanercept (unspecified underlying disease), as reported to the FDA adverse event report system from January 1998 to September 2002, with the number of people using these medications in the period.	The authors identified the occurrence of 37 cases of histoplasmosis in 197,000 patients using infliximab in the period (18.78 infections/100,000 patients treated) and 3 cases of histoplasmosis in 113,000 patients using etanercept (2.65 infections/100,000 patients).
Schiff MH et al Safety analyses of adalimumab (HUMIRA) in global clinical trials and US postmarketing surveillance of patients with rheumatoid arthritis^[21]^	2006	Analysis of safety data of patients with rheumatoid arthritis treated with adalimumab from randomized controlled trials, open-label extensions, phase IIIb open-label trials, and postmarketing spontaneous reports of adverse events in the US, from December 2002 to April 2005.	There were 4 cases of histoplasmosis reported, all in endemic areas, in 10,050 patients identified (incidence rate of 39.80 cases/100,000 patients)
Pérez-Sola MJ et al BIOBADASER Study Group. Infections in patients treated with tumor necrosis factor antagonists: incidence, etiology and mortality in the BIOBADASER registry^[22]^	2011	Analysis of episodes of infection of the Spanish registry BIOBADASER, a national drug safety registry of patients with rheumatic diseases treated with TNF-ɑ inhibitors, from February 2000 to January 2006.	In this group of 6969 patients on TNF-ɑ inhibitors, no case of histoplasmosis was reported.
Titton DC et al Brazilian biologic registry: BiobadaBrasil implementation process and preliminary results. Rev Bras Reumatol. 2011 Mar-Apr;51(2):152–60.^[23]^	2011	Report on the clinical data of the implementation process of the BiobadaBrasil registry, a national registry of patients with rheumatic diseases treated with TNF-ɑ inhibitors developed in 15 centers in Brazil, from April 2009 to February 2010.	In the 10-mo follow-up period of the 750 patients using immunobiological drugs, mostly TNF-ɑ inhibitors (mostly Infliximab), there was no record of cases of histoplasmosis.
Seminerio JL et al Infliximab for Crohn disease: the first 500 patients followed up through 2009^[24]^	2013	Analysis of the medical records of 492 unselected patients treated with infliximab for Crohn Disease at Mayo Clinic, from October 1998 to October 2002, with follow-up through September 2009.	In 492 CD patients treated with infliximab in that period, with follow-up to 2009, there were 3 cases of histoplasmosis (calculated incidence rate of 609.76 cases/100,000 patients).
Baddley JW et al Non-viral opportunistic infections in new users of tumor necrosis factor inhibitor therapy: results of the SAfety Assessment of Biologic ThERapy (SABER) study^[25]^	2014	A retrospective cohort study combining data from 1998 through 2007 from 4 large US data systems (Kaiser Permanente Northern California, 2 pharmaceutical assistance programs for the elderly, Tennessee Medicaid, and US Medicaid/Medicare programs), comparing non-viral infection rates of new users of TNF-ɑ inhibitors to non-biologic drugs.	In a cohort of 33,324 patients with RA or IBD who started the use of TNF-ɑ inhibitors in the analyzed period, there were 9 cases of histoplasmosis (calculated incidence rate of 27.01 cases/100,000 patients).
Kimball AB et al OBSERVE-5: observational postmarketing safety surveillance registry of etanercept for the treatment of psoriasis final 5-yr results. J Am Acad Dermatol.^[26]^	2015	Analysis of safety data from OBSERVE-5 (a 5-yr FDA-mandated multicenter, observational surveillance registry of psoriasis patients), with data report of patients treated with etanercept in 375 sites (37 Canada, 338 US).	In this group of 2510 patients on etanercept, no case of histoplasmosis was reported.
McAuliffe ME et al Occurrence of adverse events among patients with inflammatory bowel disease in the HealthCore Integrated Research Database^[27]^	2015	A retrospective cohort study of 33,386 patients with IBD in the HealthCore Integrated Research Database (HIRD - an insured US population database) between January 2004 and January 2011, analyzing safety data of patients with mild and moderate to severe IBD.	There were 3 cases of histoplasmosis in 3348 patients treated with TNF-ɑ inhibitors in the period described (calculated an incidence rate of 89.61 cases/100,000 patients).
Kay J et al Five-year Safety Data from 5 Clinical Trials of Subcutaneous Golimumab in Patients with Rheumatoid Arthritis, Psoriatic Arthritis, and Ankylosing Spondylitis^[28]^	2016	Safety data from 5 large multicenter trials of patients with rheumatic diseases (RA, Psoriatic Arthritis, and Ankylosing Spondylitis) treated with golimumab.	There were 3 cases of histoplasmosis in 2228 patients treated with golimumab (calculated incidence rate of 134.65 cases/100,000 patients).
Salt E et al Risk Factors for Targeted Fungal and Mycobacterial Infections in Patients Taking Tumor Necrosis Factor Inhibitors^[29]^	2016	A case-control study using deidentified patient health information from a large database set of commercially insured US population, that identified 30,772 patients treated with TNF-ɑ inhibitors for autoimmune diseases (unspecified underlying disease), from January 2007 to December 2009.	The authors identified 95 patients using TNF-ɑ inhibitors who had histoplasmosis in this group of 30,772 patients (calculated incidence rate of 308.72 cases/100,000 patients).
Lichtenstein GR et al Infliximab for Crohn Disease: More than 13 yr of real-world experience^[30]^	2018	Evaluation of long-term safety outcomes from an observational, multicenter, long-term North American registry of patients with CD in treatment infliximab, from July 1999 to September 2012. The infliximab-treated patients were followed for 20,971 patient-years (PYs).	There were 2 cases of histoplasmosis (both from the US) in patients from a group of 3440 single patients using infliximab for IBD evaluated (authors described incidence rate of 0.00/100 PYs, calculated incidence rate of 58.14 cases/100,000 patients).

CD = Crohn Disease, FDA = Food and Drug Administration, IBD = inflammatory bowel disease, RA = rheumatoid arthritis.

### 3.2. Occurrence of histoplasmosis in patients treated with TNF-α inhibitors

Although the relationship between therapy with TNF-α inhibitors and the increased occurrence of granulomatous diseases has been gradually perceived and understood over the last quarter of a century, there are still few studies available for estimating risk rates in this population. The first major study to estimate the incidence rate of histoplasmosis in people taking TNF-ɑ inhibitory drugs was published by Wallis et al in 2004, examining the infectious granulomatous complications of this therapy.^[31]^ The following year, the same author published a new estimate that relied on events reported through the FDA Adverse Event Reporting System (AERS) from January 1998 through September 2002, in which the incidence of histoplasmosis was calculated as 18.78 cases per 100,000 patients treated with infliximab and 2.65 cases per 100,000 patients treated with etanercept, based on the number of US patients receiving these medications in September 2002 ^[20]^. This is still the incidence rate used as a reference in many publications on the subject to date. On the first estimate, the incidence rate in infliximab users was calculated as 16.7/100,000, and in etanercept users, it was the same.^[31]^ Histoplasmosis was the second most frequent granulomatous disease in people who received a TNF-ɑ inhibitor, second only to tuberculosis, which had an incidence of 129.44 infections per 100,000 infliximab users.

Studies with sufficient data for calculating the incidence rate included a prospective cohort conducted by Seminerio et al, in a follow-up study carried out for more than a decade until 2009. The authors examined the records of 492 US patients who used infliximab for Crohn Disease (CD), of which 3 had histoplasmosis, that is, an estimated incidence rate of 609.76 cases/100,000 patients.^[24]^ Moreover, our review identified large retrospective cohorts and case-control studies, which allowed the calculation of incidence rates of histoplasmosis ranging from 27.01/100,000 (in US patients with RA or IBD treated with a TNF-ɑ inhibitor 1998 through 2007^[25]^), to 89.61/100,000 (in US patients treated with a TNF-ɑ inhibitor for IBD from January 2004 through January 2011^[27]^), and up to 308.72/100,000 (in US patients followed by use of any TNF-ɑ inhibitor from January 2007 through December 2009^[29]^).

Histoplasmosis also appears to be a treatment complication in some safety databases of TNF-α inhibitory drugs. Schiff et al found 4 cases of histoplasmosis, all occurring in endemic areas, in 10,050 patients using adalimumab from December 2002 to April 2005 from a US database, which allowed to estimate an incidence rate of 39.80 cases/100,000 patients.^[21]^ Later, in a 5-year safety data report from 5 clinical trials involving golimumab, there were 3 cases of histoplasmosis among 2228 patients receiving therapy, thus admitting calculation of the incidence rate of 134.65 cases/100,000 patients.^[28]^ In other studies, histoplasmosis was found to be a much less frequent complication. Recently, Lichtenstein et al published the results of an extensive prospective observational registry evaluation of North American patients with CD who were treated with infliximab between July 1999 and September 2012.^[30]^ There were only 2 cases of histoplasmosis among the 3440 patients followed in that interval^[30]^.

### 3.3. Meta-analysis of the results

**Figure 2.**
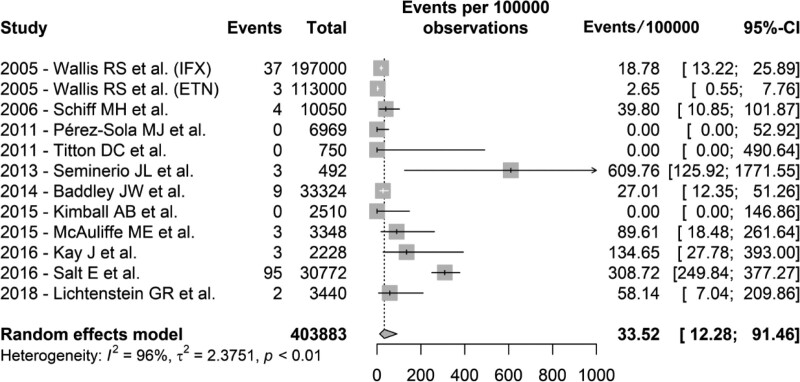
Forest plot of the meta-analysis of the selected studies. Events in cases of histoplasmosis per 100,000 patients on treatment. ETN = etanercept, IFX = infliximab.

A meta-analysis of the studies that met the inclusion criteria was performed. Since Wallis et al presented 2 independent rates for populations using either infliximab (IFX) or etanercept (ETN), with results quite different from each other, each incidence was considered as the sole factor in the calculation.^[20]^ The incidence rate of histoplasmosis was 33.52 cases per 100,000 patients treated with TNF-α inhibitors (95% CI 12.28–91.46). The data in a forest plot is summarized in Figure 2.

### 3.4. Differences by geographic location in the incidence of histoplasmosis

Nine of the eleven studies included in the analysis addressed patients who were followed up at centers located in North America, mostly in the USA and Canada, and the remaining studies were conducted in Spain^[22]^ and Brazil.^[23]^ Interestingly, a 2011 report on the preliminary data of the implementation process of a national registry of patients with rheumatic diseases, the BiobadaBrasil, conducted in 15 centers in Brazil, a country where histoplasmosis is endemic, found no cases of the disease among 750 patients treated with biological drugs, mostly TNF-ɑ inhibitors, in a 10-month follow-up.^[23]^ However, it is possible that the disease was underdiagnosed, since detection of *H capsulatum* by molecular methods, such as antigen testing, was not available in Brazil at that time. Likewise, there were no cases of histoplasmosis recorded in the BIOBADASER, a large safety registry of a cohort of patients with RA treated with TNF-α inhibitors followed in Spain, a country where histoplasmosis is not considered an endemic mycosis.^[22]^

### 3.5. Analysis by underlying disease and class of TNF-inhibiting agent

**Figure 3.**
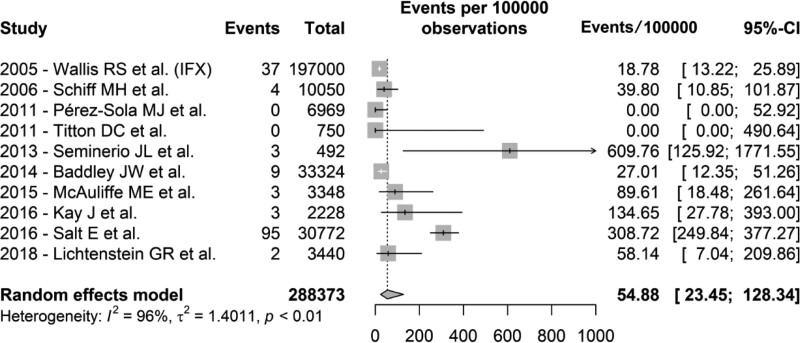
Forest plot of the meta-analysis of the selected studies, excluding those exclusively involving etanercept. Events in cases of histoplasmosis per 100,000 patients on treatment. IFX = infliximab.

**Figure 4.**
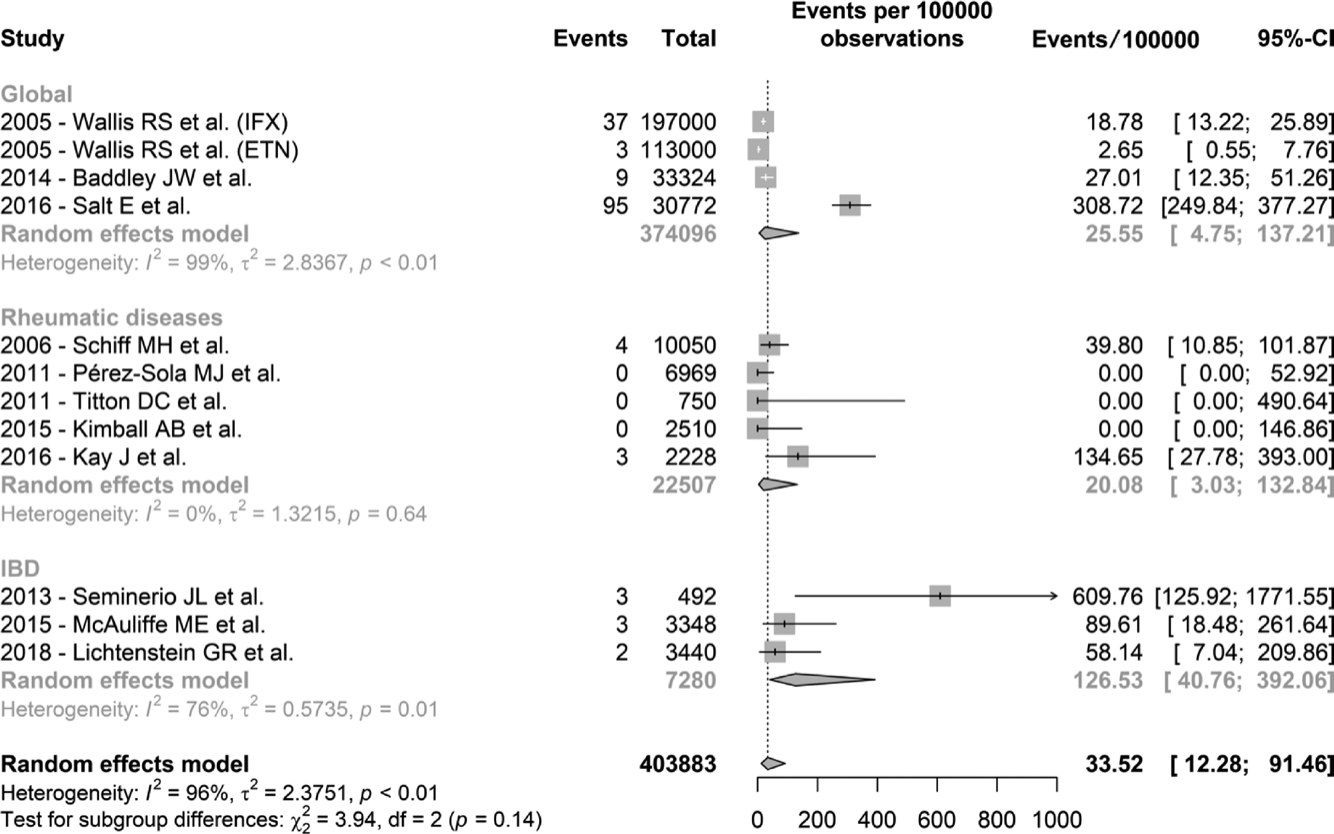
Forest plot of the meta-analysis of the selected studies stratified by inflammatory bowel disease, rheumatic diseases, and a global group (unspecified underlying disease). Events in cases of histoplasmosis per 100,000 patients on treatment. ETN = etanercept, IFX = infliximab.

TNF-α inhibition therapy can be performed with monoclonal antibody agents (infliximab, adalimumab, certolizumab pegol, and golimumab), or with a circulating receptor fusion protein (etanercept). The review published in 2005 by Wallis et al highlighted a difference between the incidence of histoplasmosis associated with the 2 drugs analyzed, much higher in patients treated with infliximab compared to etanercept. Most of the articles in the meta-analysis assessed a monoclonal antibody, while in some there was no agent differentiation. Etanercept was evaluated exclusively in an analysis of safety data from a multicenter study involving 2510 patients published in 2015, where there were no records of cases of histoplasmosis.^[26]^ An analysis was performed excluding the 2 groups in which etanercept was the only TNF-α inhibitor used, and the incidence of histoplasmosis was 54.88 cases per 100,000 patients treated (95% CI 23.45–128.34, Figure 3). Additionally, a subgroup analysis was performed regarding the incidence of histoplasmosis according to the underlying disease evaluated in each article present in the meta-analysis, when described. Figure 4 presents an analysis of the groups stratified between inflammatory bowel disease, rheumatic diseases, and a global group, in which the underlying condition of the patients treated with TNF-α inhibitors was not discriminated in the article.

## 4. Discussion

The recognition of TNF-α as a central mediator of several inflammatory processes has led to the investigation of TNF-α inhibitory agents as a therapeutic option for the treatment of inflammatory diseases, especially autoimmune conditions, such as rheumatic diseases and IBD.^[32]^ The US FDA approved infliximab (*Remicade®*) as the first TNF-α inhibitor drug in October 1998, followed by etanercept (*Enbrel®*) in November 1998. Since then, additional TNF-α inhibitory drugs, such as adalimumab (*Humira®*), certolizumab pegol (*Cimzia®*), and golimumab (*Simponi®*) have also received approval. However, despite pre-approval placebo-controlled clinical trials that do not indicate an increased risk of infectious complications, the potential for adverse effects related to infections remained a significant concern because of the immunosuppressive nature of these agents.

After 2 case reports of histoplasmosis associated with the use of infliximab,^[33,34]^ the first case series of severe disease caused by *H capsulatum* in patients using TNF-α inhibitors was published in 2002 by Lee et al, in which ten infections were associated with the treatment.^[35]^ Tsiodras et al (2007) examined the reports of patients on any TNF-ɑ blocking therapy presenting with invasive fungal infections published on the MEDLINE and PubMed platforms between 1966 and June 2007.^[17]^ Of the 281 cases of invasive fungal disease reported in this population, the most prevalent invasive fungal infection was histoplasmosis (84 episodes). A black box warning was issued by the FDA in 2008, after reviewing 240 reports of histoplasmosis in patients treated with TNF-α inhibitors, most from areas where *H capsulatum* is endemic, and in at least 21 of these reported cases the infection was not diagnosed until a late stage.^[36]^ Even though these studies were valuable in establishing the notion of a higher risk for histoplasmosis in patients on TNF-blocking therapy, most subsequent publications do not provide all the data that would allow the calculation of an incidence rate. We sought to locate studies that provided the necessary parameters to accurately estimate the risk of illness in these patients.

Studies pooled in the meta-analysis were considered heterogeneous in several ways. In addition to a noticeable variation in the number of patients analyzed, ranging from a few hundred to tens of thousands, some studies focused on several diseases, while others solely investigated certain pathologies, such as IBD or RA. Likewise, some articles addressed single drugs, whereas others evaluated TNF-α inhibitors as a class. Finally, the grouped articles used varied methodologies, including medical records review, prospective cohorts, and case-control studies, and it is also difficult to account for the impact of concomitant medications on the results. Even so, the calculated incidence rate of 33.52 cases of histoplasmosis per 100,000 treated patients is a considerable figure, especially when compared to the traditional reference of 18.78/100,000 patients, an estimate of almost 20 years ago^[20]^ (Fig. 2). It is interesting to note that most studies identified a low or moderate incidence of histoplasmosis, with only a few demonstrating very high rates of incidence. This is likely related to research involving patients from endemic or hyperendemic areas. For example, the highest estimated incidence of histoplasmosis was identified in a study conducted at the Mayo Clinic in Rochester, MN, located near the Mississippi River in the Upper Midwest of the US.^[24]^ Similarly, in the case-control study that identified more than 308 cases per 100,000 patients, the majority of people diagnosed with histoplasmosis were from the US Midwest.^[29]^

The sensitivity analysis excluding data from the 2 groups known to be exclusively using etanercept, a TNF-ɑ inhibitor with arguably lesser immunosuppressive potential, demonstrated that the incidence rate of histoplasmosis increased to 54.88/100,000 patients (Fig. 3). This difference in the occurrence of histoplasmosis between different types of TNF-α inhibitor drugs was a tendency suggested since the first reports. Of the 10 cases presented by Lee et al in 2002, 9 patients were diagnosed using infliximab and only one using etanercept.^[35]^ Subsequent studies with a larger number of patients, such as the review published in 2005 by Wallis et al, showed similar trends.^[20]^ Of the 281 cases of invasive fungal infection presented by Tsiodras et al in 2007, 80% used infliximab, 16% used etanercept, and 4% used adalimumab.^[17]^ A single-drug study published in 2013 included in the meta-analysis, on a cohort of patients with IBD treated with infliximab, also showed a high incidence of histoplasmosis.^[24]^ In contrast, in the safety data analysis from a multicenter study involving 2510 patients using etanercept published in 2015, there were no records of cases of histoplasmosis.^[26]^ Distinctions between the molecular structures and mechanisms of action are probably involved in these observed differences, especially concerning etanercept. Different from other agents of the class, which are monoclonal antibodies, etanercept is a soluble TNF-inhibitor factor with a recognized less extensive immunosuppressive effect than other drugs, such as infliximab.^[37]^

Other circumstances that certainly influence diagnosis rates of *Histoplasma* infection are the baseline disease of the patient using immunomodulatory therapy and associated medications. Some conditions require more aggressive immunosuppressive therapy for control. For example, in the cohort of 492 patients with IBD evaluated by Seminerio et al, concomitant with infliximab therapy, 79% used corticosteroids, while 76% used a third agent (Azathioprine, 6-Mercaptopurine or Methotrexate).^[24]^ The concomitant use of other immunosuppressants generates a confounding factor when assessing the individual contribution to the risk of a TNF-α inhibitor. Furthermore, some recent studies suggest that other common immunosuppressive agents, such as corticosteroids, may contribute as much or even more than biological agents to the susceptibility to histoplasmosis.^[38]^ Most of the studies grouped in the meta-analysis did not contain sufficient information to allow a detailed analysis regarding associated immunosuppressants.

Heterogeneity was controlled in the subgroup analyses shown in Figure 4. This effect was observed especially in those studies that evaluated rheumatic diseases (RA, psoriatic arthritis, spondylitis), a group that became very homogeneous and presented a calculated incidence of 20.08 cases of histoplasmosis per 100,000 patients treated with a TNF-α inhibitor, similar to the traditional estimate. In the group in which the therapy was evaluated globally, when data on the underlying disease were not available, and included the broad population studies, the incidence was 25.55 cases/100,000 patients. The most notable difference appears when observing the incidence in patients with IBD, with 126.53 cases/100,000 patients treated with a TNF-α inhibitor, 6 times higher than that found in patients with rheumatic disease and almost 4 times higher than the overall incidence found in the meta-analysis. It is not clear whether this difference could be due to the effects of the baseline condition of the patients or the need for a greater degree of immunosuppression.

The risk of infection also has a crucial influence on the region of origin of the patient groups, as the possibility of the occurrence of histoplasmosis becomes more remote in places where the disease is not endemic. Although *H capsulatum* is distributed worldwide except in Antarctica, histoplasmosis is considered endemic in specific areas, including the western US, South America, northern Africa, and part of Asia, Europe, and Oceania.^[3,39,40]^ The impact of epidemiological association can be observed in the analysis of safety data on patients being treated for rheumatic diseases in centers in Spain published by Pérez-Sola et al in 2011, in which none of the 6969 patients using TNF-α inhibitors evaluated over 6 years had histoplasmosis.^[22]^ Most studies that met the inclusion criteria of this review were carried out in centers in the United States and Canada, and most of them presented a considerable number of diagnoses of histoplasmosis.

The limitations of our research include the small number of available studies on this topic. Many reports and case series focusing on the complications of treatment with immunomodulatory drugs are available, but there are still few of a larger observational nature. Even relevant studies, such as the retrospective review of 98 cases of histoplasmosis in TNF-α inhibitor users published by Vergidis et al in 2015, the largest retrospective analysis of individual cases of this sort, lacked the necessary information regarding the source population required in our selection criteria for calculating the incidence rate.^[19]^

Notably, published research on histoplasmosis is scarce involving centers in Latin America. Indeed, there are still a small number of robust studies regarding this region, even though histoplasmosis is currently considered the most prevalent endemic mycosis on the Latin American continent.^[41]^ A high prevalence of this infection in people living with HIV has already been demonstrated in studies carried out in Brazil, French Guiana, and Guatemala, especially in those with a CD4 cell count < 50/mm^3^.^[42–45]^ Although the burden of disseminated histoplasmosis in people with AIDS has gained relevance in recent years, histoplasmosis-related morbidity in patients treated with biologics in South America remains understudied.

## 5. Conclusion

The occurrence of opportunistic infections during treatment with immunosuppressive therapies is a significant concern for healthcare centers. However, the availability of materials in medical literature on some topics is limited. To the best of our knowledge, this is the first systematic review and meta-analysis on the incidence of histoplasmosis in patients using TNF-α inhibitory therapies. The incidence of histoplasmosis in the described population may be significantly higher than that in the general population, especially in those using monoclonal antibodies or in treatment for IBD. There is an urgent need for an updated population study designed to assess the current rate of this fungal infection in users of TNF-α inhibitors, as well as to understand possible factors contributing to this increased incidence, such as associated immunosuppressive medications or a broader use in people more vulnerable in endemic areas. Furthermore, there is an alarmingly low number of studies outside North America on this type of opportunistic infection, notably in Central and South America. This topic deserves to be briefly addressed, as the number of patients being treated for autoimmune diseases with TNF-α inhibitory agents in this region is increasing.^[46]^ We hope that the availability of diagnostic methods for histoplasmosis will facilitate studies on HIV-negative populations in these regions.

## Acknowledgements

Data analysis was reviewed through a consultation with a biostatistician, from Hospital de Clinicas de Porto Alegre, and supported by FIPE-HCPA.

## Author contributions

**Conceptualization:** Alessandro C. Pasqualotto, Diego Rodrigues Falci.

**Data curation:** Murillo M. Cipolat, Débora R.R. Rodrigues, Claiton V. Brenol, Alessandro C. Pasqualotto, Diego Rodrigues Falci.

**Formal analysis:** Murillo M. Cipolat, Diego Rodrigues Falci.

**Funding acquisition:** Diego Rodrigues Falci.

**Investigation:** Murillo M. Cipolat, Débora R.R. Rodrigues, Claiton V. Brenol, Alessandro C. Pasqualotto, Diego Rodrigues Falci.

**Methodology:** Claiton V. Brenol, Alessandro C. Pasqualotto, Diego Rodrigues Falci.

**Project administration:** Diego Rodrigues Falci.

**Supervision:** Claiton V. Brenol, Alessandro C. Pasqualotto, Diego Rodrigues Falci.

**Writing – original draft:** Murillo M. Cipolat, Alessandro C. Pasqualotto, Diego Rodrigues Falci.

**Writing – review & editing:** Murillo M. Cipolat, Claiton V. Brenol, Alessandro C. Pasqualotto, Diego Rodrigues Falci.
